# Percent Predicted vs. Absolute Six-Minute Walk Distance as Predictors of Lung Transplant-Free Survival in Fibrosing Interstitial Lung Diseases

**DOI:** 10.1007/s00408-024-00748-5

**Published:** 2024-09-20

**Authors:** Umberto Zanini, Jane Ding, Fabrizio Luppi, Karina Kaur, Niccolò Anzani, Giovanni Franco, Giovanni Ferrara, Meena Kalluri, Marco Mura

**Affiliations:** 1 Department of Medicine and Surgery, University of Milan Bicocca, Respiratory Unit, Fondazione IRCCS San Gerardo Dei Tintori, Monza, Italy; 2 Division of Respirology, Western University, London, Canada; 3 Division of Pulmonary Medicine, University of Alberta, and Alberta Health Services, Edmonton, Canada

**Keywords:** Fibrosing interstitial lung diseases, Six-minute walk test, Exercise capacity, Percent predicted, Reference equation, Transplant-free survival

## Abstract

**Introduction:**

Fibrosing interstitial lung diseases (ILDs) often progress despite treatment and become life-threatening, with lung transplant (LTx) remaining the only curative option. Six-minute walk distance (6MWD) is increasingly recognized as reliable predictor of clinical course, especially when longitudinally considered. The use of reference equations to express 6MWD as percent predicted (6MWD%) has not been previously studied in fibrosing ILDs. We sought to investigate whether the prognostic power of 6MWD% is superior to that of 6MWD expressed in meters (6MWD-m).

**Methods:**

A retrospective, multicenter cohort analysis was conducted on both idiopathic pulmonary (IPF) and non-IPF fibrosing ILD patients. Patients were divided into a discovery (*n* = 211) and a validation (*n* = 260) cohort. Longitudinal changes of 6MWD% and lung function parameters were simultaneously considered. LTx-free survival at 3 years from baseline was the endpoint. Competing risks of death and LTx were considered.

**Results:**

Baseline 6MWD% and its longitudinal changes were significant predictors of LTx-free survival and independent from lung function variables. In both cohorts, on multivariate cox proportional hazard regression analysis, receiver operating characteristics analysis and Kaplan–Meier estimates, 6MWD% was consistently, but only slightly superior to 6MWD-m as a predictor of LTx-free survival.

**Conclusion:**

6MWD% has only a slight, yet detectable advantage over 6MWD-m as a predictor of survival in fibrosing ILDs. Utilizing 6MWD% may aid in risk stratification, treatment monitoring, and LTx timing optimization. However, available reference equations do have predicting limitations. Refined predictive equations and standardizing reporting practices are therefore needed to further enhance the clinical utility of 6MWD% in fibrosing ILDs.

**Supplementary Information:**

The online version contains supplementary material available at 10.1007/s00408-024-00748-5.

## Introduction

Fibrosing interstitial lung diseases (ILDs) are a heterogeneous group of different ILDs with variable courses and prognoses [1]. Idiopathic pulmonary fibrosis (IPF) is the most common, and unfortunately most progressive type of fibrosing ILD, with a mean survival of 3–5 years [2]. A significant proportion of patients with other types of fibrosing ILD can also develop progression, despite optimal immunomodulatory treatment [3].

Given the high mortality of progressive, fibrosing ILD [4], there currently is a great interest in recognizing and validating the most reliable clinical predictors of clinically significant progression in fibrosing ILD. Such patients may be started on anti-fibrotic therapy [5] and, if eligible, assessed for lung transplantation (LTx), which remains the only curative option [6].

Early detection of progression is crucial for starting timely treatment, assessing the need for a LTx, and making accurate prognostications. Traditionally, clinicians have been relying on lung function decline and radiological worsening on high-resolution chest CT scan (HRCT) to identify progression [7]. The American Thoracic Society proposed clinical, functional, and radiographic parameters within 12 months as criteria to define progression [8].

Pulmonary function tests (PFTs) however may not capture the full impact of the fibrosing disease on the patient’s functional status [9]. Six-minute walk distance (6MWD) is a recognized predictor of survival in IPF [10, 11]. We recently demonstrated that a longitudinal decline of 6MWD predicts LTx-free survival independently from lung function decline also in patients with non-IPF fibrosing ILDs [12]. 6MWD was in fact proposed as a primary endpoint in ILD research, as it may represent a more global measurement of functional capacity and exercise tolerance than PFTs [9]. The six-minute walk test (6MWT) is also simple, inexpensive and reproducible [13, 14].

Almost all previous studies investigating the role of 6MWD in ILDs considered only 6MWD expressed in meters (6MWD-m), but this may not account for expected physiological differences based on age, sex, height, and weight. In fact, ILD patients are characterized by older age and higher burden of comorbidities than the average population [15, 16].

Reference equations for predicted 6MWD were derived from accounting for these factors and can be used to obtain individual 6MWD percent of predicted value. Assessing the patient’s functional capacity and disease trajectory with greater accuracy may provide more reliable insights into their condition [17]. The reference equations of Enright and Sherill [17] are widely used in clinical practice to obtain a percent predicted 6MWD (6MWD%). However, 6MWD% has not been investigated as a predictor of survival in large and diverse cohorts of patients with fibrosing ILD.

We hypothesized that 6MWD% may be a better predictor of survival in fibrosing ILDs than 6MWD-m. Therefore, the aim of this multi-centre study was to compare the predictive power of 6MWD-m and 6MWD% towards LTx-free survival in patients with both IPF and non-IPF fibrosing ILDs.

## Methods

### Study Design

This study is a retrospective multi-center cohort analysis, including patients with fibrosing ILDs evaluated and followed at 3 different tertiary referral centers (London [ON], Edmonton, Canada, and Monza, Italy). The population studied included a discovery cohort (London) and a validation cohort (Edmonton and Monza), which was tested to confirm the findings of the discovery cohort. Data were collected at baseline on initial consultation, and longitudinal follow-up data were collected at 1 year (± 3 months) from baseline. Diagnosis was made based on multidisciplinary discussion (MDD) at the local institution, with available clinical, radiologic, and pathologic information. Clinical characteristics evaluated included age, gender, body mass index (BMI), smoking history, specific type of ILD diagnosed at MDD, and treatments used. Pulmonary function tests (forced vital capacity [FVC] and diffusing lung capacity for carbon monoxide [DLCO]) and 6MWD were obtained according to the ERS/ATS guidelines [18–20]. 6MWD% was calculated according to the reference equations of Enright and Sherrill [17]. The primary endpoint was LTx-free survival at 3 years from baseline.

Both patients with IPF and non-IPF fibrosing ILD were included. Data of the non-IPF fibrosing ILD subjects from the discovery and validation cohorts were previously published [12].

The study was approved by the Research Ethics Boards of Western University (n.103186), University of Alberta (n.00082981) and University of Milan “Bicocca” (n.1538).

### Statistical Analysis

The distribution of data was assessed using the Shapiro–Wilk test. Comparisons between the discovery and validation cohorts were conducted using unpaired t-test, Mann–Whitney U-test, or Chi-square test, as appropriate. Values are presented as mean ± standard deviation. Receiver operating characteristic (ROC) analysis was performed to identify an optimal 6MWD% cutoff value to predict the endpoint. For 6MWD-m, the cutoff of ≥ −24 m decline was used, based on our recent study [12]. Competing risk regression analysis (Fine-Gray method) was used to identify the significance of variables in predicting the risk of death before LTx. To account for low exercise capacity at baseline in patients who passed away before their 1-year reassessment, both baseline and longitudinal changes of 6MWD were considered in multivariate regression analysis. Survival curves were generated using the Kaplan–Meier method and compared using the log-rank test. P-values < 0.05 were considered significant. JMP (SAS Institute, Cary, NC), MedCalc (MedCalc, Mariakerke, Belgium) and Stata (StataCorp, College Station, TX) statistical softwares were used.

### Results

The discovery cohort consisted of 211 patients and the validation cohort of 260 patients. The recruitment process of the 2 cohorts is detailed in Supplemental Fig. 1. Demographic data are summarized in Table 1. There were no significant differences in demographics between the discovery and validation cohorts. However, there were differences in the proportion of some ILD subtypes (IPAF, NSIP, sarcoidosis). Patients in the validation cohort had significantly higher DLCO at baseline and at 1 year, although there was no difference in FVC, 6MWD-m, or 6MWD%. The mean follow-up was 42 ± 24 months in the discovery cohort and 35 ± 25 months in the validation cohort. There were also significant differences in the use of immunosuppressive and anti-fibrotic therapy between the 2 cohorts, reflecting different practices. The combined incidences of death/LTx at 3 years from baseline were 35% and 20% (p < 0.001) in the discovery and validation cohort, respectively (Table 1).

**Fig. 1:**
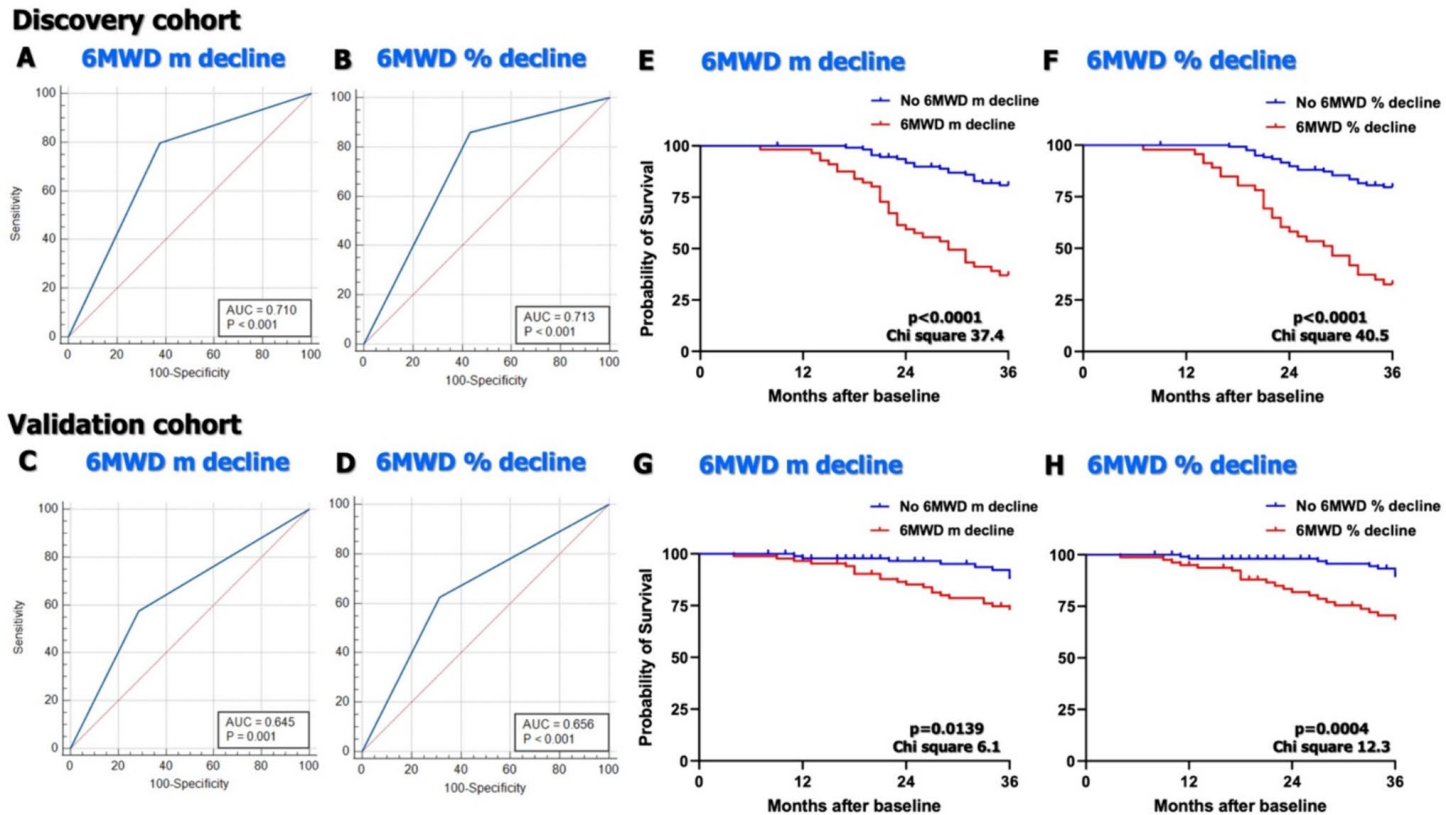
Receiver operating characteristics (c-statistics) and Kaplan–Meier survival analysis for 3-year LTx-free survival. **A**, **B**: 6MWD-m and 6MWD% longitudinal decline in the discovery cohort. **C**, **D**: 6MWD-m and 6MWD% longitudinal decline in the validation cohort. **E**, **F**: Kaplan–Meier analysis for 6MWD-m and 6MWD% longitudinal decline in the discovery cohort. **G**, **H**: Kaplan–Meier analysis for 6MWD-m and 6MWD% longitudinal decline in the validation cohort

**Table 1:** Demographic, clinical, and functional characteristics of patients included in the study

	Discovery cohort (London) *n* = 211	Validation cohort (Edmonton, Monza)* N* = 260	p-value
Age (years)	70 ± 10	68 ± 12	n.s
Male	127 (60%)	159 (61%)	n.s
Diagnosis			
IPF	106 (50%)	122 (47%)	n.s
CTD-ILD	36 (17%)	29 (11%)	n.s
Unclassifiable	27 (13%)	39 (15%)	n.s
IPAF	13 (6%)	2 (0.8%)	0.001
HP	11 (5%)	9 (4%)	n.s
NSIP	9 (4%)	42 (16%)	< 0.001
Drug-related	2 (1%)	1 (0.4%)	n.s
Sarcoidosis	1 (0.5%)	8 (3%)	0.040
Other	6 (3%)	8 (3%)	n.s
BMI (kg/m^2^)	30 ± 6	30 ± 6	n.s
FVC (% pred)	77 ± 21	77 ± 18	n.s
FVC (% pred) at 1 year	75 ± 22	78 ± 19	n.s
DLCO (% pred)	48 ± 17	54 ± 16	< 0.001
DLCO (% pred) at 1 year	44 ± 19	51 ± 16	0.002
6MWD (m)	380 ± 113	397 ± 113	n.s
6MWD (m) at 1 year	377 ± 117	390 ± 119	n.s
6MWD (% pred)	81 ± 24	86 ± 25	n.s
6MWD (% pred) at 1 year	80 ± 23	84 ± 24	n.s
Follow-up (months)	42 ± 24	35 ± 25	n.s
Antifibrotic therapy	93 (43%)	143 (55%)	0.01
Immunosuppressive therapy	79 (37%)	49 (19%)	< 0.001
Alive/dead/LTx*	140/55/19	207/51/2	< 0.001

*IPF* idiopathic pulmonary fibrosis. *CTD-ILD* connective tissue disease-associated interstitial lung disease. *HP* hypersensitivity pneumonitis. *IPAF* interstitial pneumonia with autoimmune features. *NSIP*: nonspecific interstitial pneumonia. BMI body mass index. *FVC* forced vital capacity. *DLCO* diffusion capacity of the lungs for carbon monoxide. *6MWD*: 6-min walk distance. *LTx* lung transplant

^a^at 3 years from baseline

Baseline 6MWD-m and 6MWD% were significant predictors of LTx-free survival in the discovery cohort, with subdistribution hazard ratios (SHR) of 0.99 and 0.98, respectively. 6MWD% was a slightly more significant predictor (p value < 0.0001 vs. 0.0036). Results were reproduced in the validation cohort (Table 2).

**Table 2:** Multivariate regression analysis. Predictors of 3-year lung transplant-free survival, 6MWD-m vs. 6MWD % pred

	Discovery cohort (London, ON)* n* = 211			Validation cohort (Edmonton, Monza)* n* = 260		
Model 1	Chi square 39.6			Chi square 14.4		
Variable	Hazard ratio	C.I	p value	Hazard ratio	C.I	p value
6MWD m baseline	0.99	0.99–0.99	0.0036	0.99	0.99–099	0.0050
6MWD 1-yr decline ≥ 24 m	5.34	3.07–9.52	< 0.0001	2.01	0.95–4.54	0.0699
Model 2	Chi square 50.6			Chi square 16.9		
Variable	Hazard ratio	C.I	p value	Hazardratio	C.I	p value
6MWD % baseline	0.98	0.96–0.99	< 0.0001	0.98	0.97–0.99	0.0259
6MWD 1-yr decline > 8%	7.53	4.20–13.72	< 0.0001	2.71	1.30–5.95	0.0074

*6MWD* 6-min walk distance. *C.I.* confidence intervals

A longitudinal decline of 6MWD-m was a strong predictor of LTx-free survival in the discovery cohort, with an SHR of 5.34 (Table 2). In terms of accuracy, the area under the curve (AUC) was 0.710, with balanced sensitivity and specificity (Fig. 1). However, a longitudinal absolute decline > 8% of 6MWD% was again a slightly stronger predictor of LTx-free survival (HR 7.53, AUC 0.713) (Table 2, Suppl. Fig. 1). Results of the Kaplan-Meer curves aligned with these analyses, again with more significant values for 6MWD% decline, compared to 6MWD-m decline (Fig. 1). Results in the validation cohort confirmed more significant values for 6MWD% compared to 6MWD-m in predicting LTx-free survival in Cox regression, ROC analyses and Kaplan–Meier curves (Table 2, Fig. 1).

We then sought to confirm the independent prognostic role of 6MWD% in combination with measures of lung function in our cohorts. In multivariate regression analyses including either FVC or DLCO, both 6MWD% and its longitudinal decline were independent and significant predictors of LTx-free survival in the discovery cohort. In the validation cohort, only the longitudinal absolute decline of 6MWD% > 8% was a significant predictor of poor outcome in combination with either FVC or DLCO (Table 3). In these models, age, gender and BMI were not retained as significant predictors of LTx-free survival.

**Table 3:** Multivariate regression analysis. Predictors of 3-year lung transplant-free survival, 6MWD % pred and lung function variables

	Discovery cohort (London, ON) *n* = 211			Validation cohort (Edmonton, Monza) *n* = 260		
Model 1	Chi square 38.7			Chi square 19.9		
Variable	Hazard ratio	C.I	p value	Hazard ratio	C.I	p value
6MWD % baseline	0.97	0.96–0.99	< 0.001	0.98	0.95–1.01	n.s
6MWD % 1-yr decline > 8%	7.45	3.47–16.02	< 0.001	4.15	1.35–12.74	0.013
FVC % baseline	0.99	0.97–1.00	n.s	0.95	0.90–0.99	0.020
FVC % 1-yr decline ≥ 5%*	2.83	1.58–5.22	< 0.001	3.50	1.20–9.86	0.018
Model 2	Chi square 29.9			Chi square 13.8		
Variable	Hazard ratio	C.I	p value	Hazard ratio	C.I	p value
6MWD % baseline	0.98	0.96–0.99	0.014	0.98	0.95–1.01	n.s
6MWD % 1-yr decline > 8%	7.02	3.01–16.35	< 0.001	3.43	1.14–10.27	0.028
DLCO % baseline	0.99	0.97–1.01	n.s	0.95	0.91–0.99	0.047
DLCO % 1-yr decline ≥ 10%*	2.09	1.05–4.15	0.036	5.31	1.55–18.16	0.008

*6MWD* 6-min walk distance. *C.I.* confidence intervals

^*^Absolute decline

Finally, we compared the prognostic power of 6MWD-m vs. 6MWD% in IPF and non-IPF patients separately, grouping the 2 cohorts together. In IPF, interestingly, 6MWD% outperformed 6MWD-m to a greater extent than in non-IPF fibrosing ILDs (Table 4).

**Table 4:** Multivariate regression analysis. Predictors of 3-year lung transplant-free survival, 6MWD-m vs. 6MWD % pred. Discovery and validation cohorts were grouped together

	IPF *n* = 228			Non-IPF ILDs *n* = 243		
Model 1	Chi square 12.4			Chi square 33.2		
Variable	Hazard ratio	C.I	p value	Hazard ratio	C.I	p value
6MWD m baseline	0.99	0.97–1.00	n.s	0.99	0.99–0.99	0.0013
6MWD 1-yr decline ≥ 24 m	1.67	0.86–3.23	n.s	4.16	2.20–8.19	< 0.0001
Model 2	Chi square 17.6			Chi square		
Variable	Hazard ratio	C.I	p value	Hazard ratio	C.I	p value
6MWD % baseline	0.99	0.97–1.00	n.s	0.98	0.96–0.99	0.0003
6MWD 1-yr decline > 8%	2.81	1.45–5.45	0.0024	5.00	2.66–9.60	< 0.0001

*6MWD* 6-min walk distance. *C.I.* confidence intervals

^a^Absolute decline

## Discussion

In this multicentre study including a heterogeneous group of fibrosing ILDs, 6MWD expressed as percent predicted was found to be only a slightly more significant predictor of LTx-free survival compared to “classic” 6MWD expressed in meters. These results were consistent across 3 different statistical analyses, and reproducible in the validation cohort. There were several significant differences between the 2 cohorts in terms of ILD subtypes, outcomes and treatment strategies. The confirmation of all findings in the validation cohorts suggest a wide applicability of the results across a diverse range of fibrosing ILDs, including both IPF and non-IPF patients. Our findings suggest a potential to use an improved exercise capacity variable in the clinical management of various types and severities of fibrosing ILDs, but also an urgent need for improved reference equation for 6MWD%.

The role of longitudinal changes of 6MWD in following the clinical course of fibrosing ILD has been increasingly recognized [21]. Although reference equations for 6MWD are available and commonly used in reporting the results of the 6MWT [22], no previous studies directly compared the prognostic power of 6MWD-m and 6MWD% across diverse groups of fibrosing ILDs. Using 6MWD% may allow for adjustments based on expected differences in demographic characteristics among patients, making it a potentially more personalized measure of exercise capacity. In a study conducted on IPF and idiopathic pleuroparenchymal fibroelastosis patients, Sato et al. showed that 6MWD is indeed influenced by factors such as age, sex, height, and weight. The authors underlined that these factors need to be considered when evaluating 6MWD outcomes in ILD patients to provide a more accurate prediction of patient prognosis [23].

Our data show a slight superiority of 6MWD% over 6MWD-m as a predictor of LTx-free survival, when used longitudinally, across different statistical approaches and diverse cohorts. Using the cutoffs for lung function (FVC and DLCO) decline recommended by the ATS [8], we were able to confirm that the simultaneous use of 6MWD% provides additional, independent predictive power towards survival. Using 6MWD% as a standardized measure may then further improve risk stratification in patients with fibrosing ILDs, identify high-risk patients, and optimize the timing for LTx. Additionally, it could help monitoring the effectiveness of treatments, informing decisions about continuing or adjusting therapy.

While using a percent predicted 6MWD would make clinical sense, the differences in prognostic power that we observed compared to 6MWD-m were overall modest. This likely due to limitations in the reliability of the reference evaluations themselves. Enright and Sherrill indicated that the predictive equations used to calculate 6MWD% were primarily derived from populations of healthy subjects [17]. It is important to note that these prediction equations only capture 60% of the variance in documented 6MWD, as the authors pointed out in their original publication [17]. The data used to develop these equations were derived from healthy subjects under 80 with a BMI under 35. BMI has an inverse linear relationship with predicted 6MWD [17]. As a result, the formula may not accurately reflect the effects of weight extremes, frailty, and advanced age, which are common characteristics in patients with fibrosing ILDs. Another intriguing and potentially confusing factor is represented by the fact that, in IPF, a higher BMI has been consistently shown to be protective against mortality [24]. Patients with a higher BMI have a lower percent predicted 6MWD. As a result, at least in the IPF subgroup, the protective effect of a higher BMI may somewhat curb the predictive power of a lower 6MWD% against mortality. On the other hand, BMI was not retained as a significant predictor of LTx survival in the multivariate cox proportional hazard regression analysis and, in fact, 6MWD% outperformed 6MWD-m to a greater extent in IPF than in non-IPF ILDs. This finding confirms a previous observation in a prospective IPF cohort [11].

Another study underlined the current limitations of 6MWD%. Balashov et al. highlighted that using the equation developed by Troosters et al. [25], 6MWD% adjusted for confounders had a better correlation with a health-related quality of life questionnaires score than 6MWD [26]. The authors argue that 6MWD often misleads the results in patients with cardiovascular disease compared to healthy subjects, due to comorbidities and demographic differences, as the standard equations for 6MWD were actually validated for healthy subjects [26]. The authors then suggested revising these equations to improve the accuracy of the outcomes’ prediction.

We considered using references other than the ones developed by Enright and Sherrill to calculate predicted 6MWD, but those are even less proficient, accounting for an even smaller portion of the physiological variance in 6MWD [27]. We advocate for the development of new predictive equations based on a wide range of patient characteristics, including old and frail patients, and including an equally wide range of BMIs, in order to improve the accuracy of 6MWD% predictions and, consequently, patient prognostication.

The present study had several strengths, including its comprehensive statistical analysis that took into account the competing risks of death and LTx. The inclusion of large, multicenter cohorts strengthened the reliability of the results. The significant prevalence of death/LTx during the observation period provided ample statistical power to investigate survival predictors. The study’s sufficiently long period of observation allowed for the assessment of both baseline and longitudinal changes in 6MWD and their impact on survival. Finally, the consistency of results among cohorts that were diverse in terms of outcomes and management, and the inclusion of both IPF and non-IPF patients both support the generalizability of the findings in fibrosing ILDs.

On other hand, the present study has also some limitations. The retrospective design carries a risk of selection and confounding bias. For the same reason, the study lacked inter-mediate assessments between baseline and 1-year follow-up. Another limitation is represented by the fact that other comorbidities and confounding factors might need to be fully accounted for, when considering both exercise capacity and outcome. Including another comorbidity score, such as the Charlson comorbidity index or the Elixhauser score, could improve the results, as reported in another study [28]. Finally, we should recognize the likelihood of a learning effect of the 6MWT in ILD patients during consecutive assessments [29].

In conclusion, our multicenter study showed that 6MWD% has a minor, yet detectable advantage over 6MWD-m as an independent predictor of LTx-free survival in a wide range of fibrosing ILDs, when considered in combination with measures of lung function. Our results support the use of a more standardized and personalized approach to the implementation of exercise capacity in the day-to-day management of fibrosing ILDs, but also calls for more accurate reference equations for 6MWD in chronic lung disease. An improved percent prediction of 6MWD will likely enhance the consistency and accuracy of the comprehensive evaluation of fibrosing ILDs across various clinical settings.

## Supplementary Information

Below is the link to the electronic supplementary material.

Supplementary file1 (DOCX 304 KB)

## Data Availability

Raw data were generated at the three centres (London [ON], Edmonton, Canada, and Monza, Italy). Derived data supporting this study’s findings are available from the corresponding author, UZ, on request.
