# Supplementary material for: Percent Predicted vs. Absolute Six-Minute Walk Distance as Predictors of Lung Transplant-Free Survival in Fibrosing Interstitial Lung Diseases
Source: Lung. 2024 Sep 20;202(6):793–800. doi: 10.1007/s00408-024-00748-5 (PMC11541322; doi:10.1007/s00408-024-00748-5)
Supplement: Supplementary file 1 — Supplementary file1 (DOCX 304 KB) [file 408_2024_748_MOESM1_ESM.docx]

**PERCENT PREDICTED vs. ABSOLUTE SIX-MINUTE WALK DISTANCE**

**AS PREDICTORS OF LUNG TRANSPLANT-FREE SURVIVAL**

**IN FIBROSING INTERSTITIAL LUNG DISEASES**

Umberto Zanini^1^, Jane Ding^2^, Fabrizio Luppi^1^, Karina Kaur^3^, Niccolò Anzani^1^, Giovanni Franco^1^, Giovanni Ferrara^3^, Meena Kalluri^3^, Marco Mura^2^

^1^Department of Medicine and Surgery, University of Milan Bicocca, Respiratory Unit, Fondazione IRCCS San Gerardo dei Tintori, ASST Monza, Italy

^2^Division of Respirology, Western University, London, Canada

^3^Division of Pulmonary Medicine, University of Alberta, and Alberta Health Services, Edmonton, Canada

**Corresponding Author**:

Umberto Zanini, MD

University of Milan-Bicocca, Fondazione IRCCS San Gerardo dei Tintori, Via Pergolesi 33, 20900 Monza, Italy

Phone: +39 039 2339040

Fax: +39 039 2336044

E-mail: [u.zanini@campus.unimib.it](mailto:u.zanini@campus.unimib.it)

**SUPPLEMENTAL MATERIAL**

**Supplemental figure 1**. Consort diagram illustrating participant selection in the study.

**
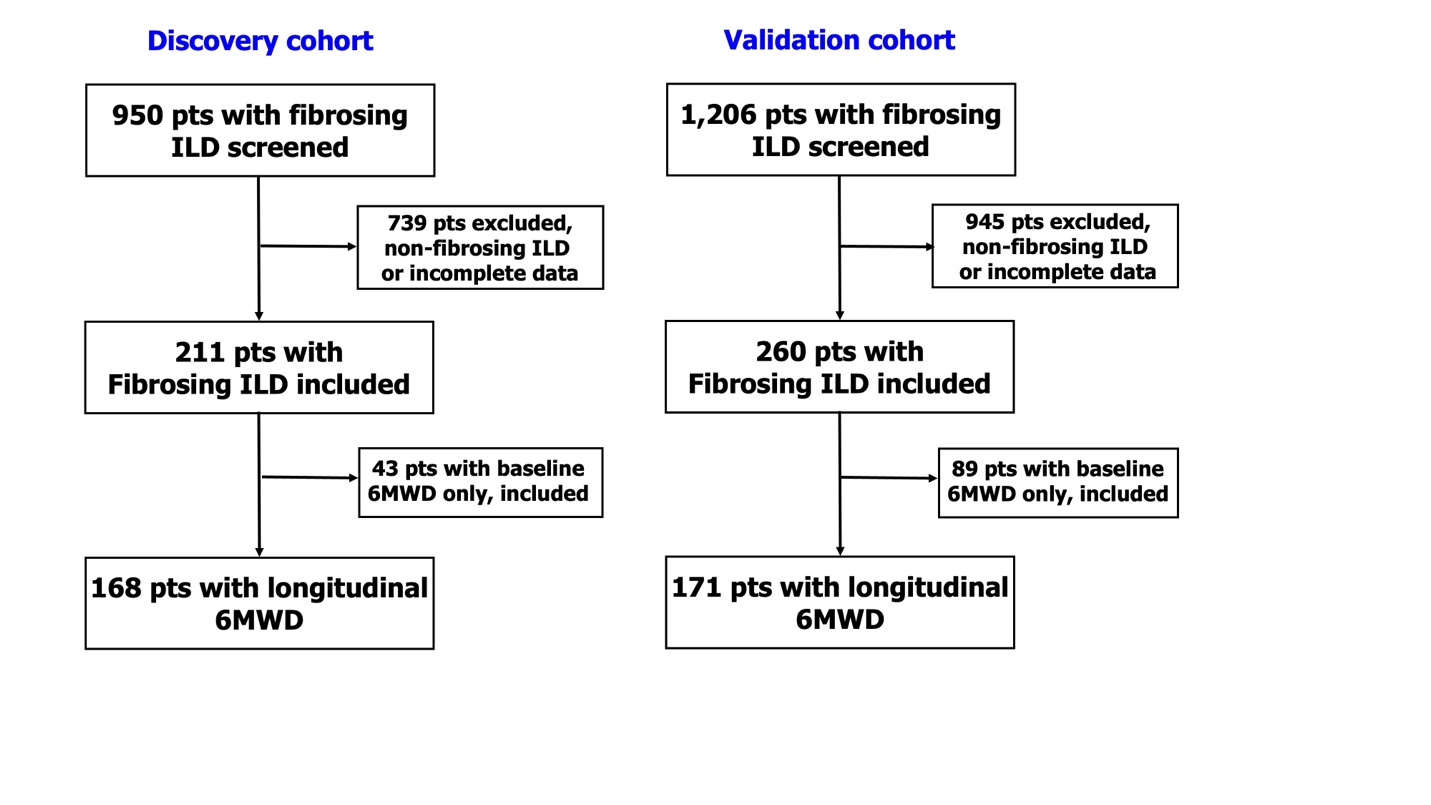
**
